# A database of traits for the ladybird species of the United Kingdom

**DOI:** 10.1038/s41597-025-05985-8

**Published:** 2025-10-29

**Authors:** Charlotte L. Outhwaite, Lauren Cocker, Richard F. Comont, Hannah J. White, Gary D. Powney, Katharine Turvey, Helen E. Roy, Peter M. J. Brown

**Affiliations:** 1 Centre for Biodiversity & Environment Research, University College London, Gower Street, London, WC1E 6BT UK; 2 Institute of Zoology, Zoological Society of London, Outer Circle, Regent’s Park, London, NW1 4RY UK; 3 Bumblebee Conservation Trust, Beta Centre, Stirling University Innovation Park, Stirling, FK9 4NF UK; 4 Centre for Ecology, Evolution and the Environment, School of Life Sciences, Anglia Ruskin University, East Road, Cambridge, CB1 1PT UK; 5 UK Centre for Ecology and Hydrology, Wallingford, OX10 8BB UK; 6 Centre for Ecology and Conservation, University of Exeter, Penryn, TR10 9FE UK

**Keywords:** Ecology, Zoology

## Abstract

Trait-based approaches have become common in ecological research as they can contribute to an understanding of ecosystem functioning and how species, communities and systems may respond to environmental change. However, trait datasets are difficult and time-consuming to compile and consequently not commonly available, particularly for insects. Ladybirds, beetles in the family Coccinellidae, are an important insect group that provide key ecosystem services, primarily through predation of pest insects such as aphids and coccids. Here, we have compiled information on species traits, ecological preferences and distribution metrics for 48 species considered resident in the United Kingdom (UK) (including the harlequin ladybird) using published sources, a recently published field guide, and biological records. Species traits may inform analyses useful for conservation purposes. This database provides researchers with access to the most up-to-date assessment of UK ladybird species, promoting research into this important insect group.

## Background & Summary

Ladybirds (Coleoptera: Coccinellidae) are generally regarded as beneficial insects, with many species playing an important role as predators of pest insects. Indeed, there are many examples of their use as biological control agents in diverse contexts globally^1^. The cultural importance and popularity of ladybirds is also notable^2,3^ and has undoubtedly contributed to the focus on them within citizen (community) science initiatives around the world^4,5^ including the UK Ladybird Survey (https://coleoptera.org.uk/coccinellidae/home).

**Fig. 1:**
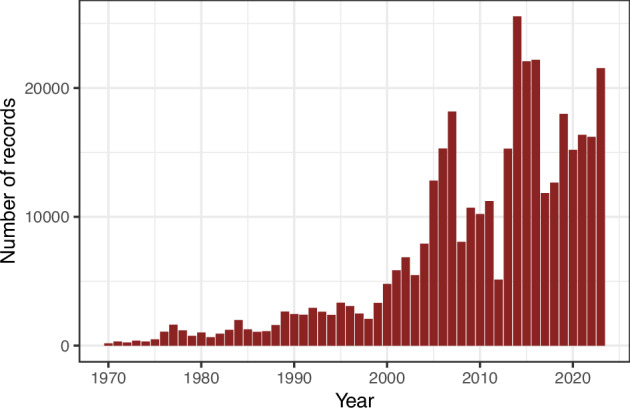
The number of records by year collected between 1970 and 2023 as part of the UK Ladybird Survey and iRecord. This figure was generated from a dataset combining records from the UK Ladybird Survey (www.coleoptera.org.uk/coccinellidae/home; provided by UK CEH) and a dataset of records from iRecord (www.irecord.org.uk; downloaded from the National Biodiversity Network Atlas - www.nbnatlas.org) where the year of the record was known and before any further processing for later use, see methods. Record numbers span from 179 records in 1970 to 21,537 records in 2023, with a peak of 25,555 records in 2014.

The UK Ladybird Survey (formerly the Coccinellidae Recording Scheme) was one of the first volunteer-led schemes to be hosted by the Biological Records Centre (part of the UK Centre for Ecology & Hydrology) which was established in 1964. The Coccinellidae Recording Scheme was established in the early 1970s and over the following decades received thousands of ladybird observations. However, following the first record of the harlequin ladybird *Harmonia axyridis* in the UK in 2004, the scheme was more widely promoted as a mass participation citizen science project called the UK Ladybird Survey. This was the first online recording scheme in the UK. The ease of recording coupled with an increase in engagement activities targeting diverse groups of people led to a substantial increase in records for all species within the family Coccinellidae (Fig. 1). The dataset compiled for *H. axyridis*, which tracked the spread of this invasive non-native species from the first record onwards, has been used to enhance understanding of biological invasions^6^. Today the UK Ladybird Survey provides volunteer-recorders with the opportunity to submit their observations for the 48 species of Coccinellidae known to be resident in the UK.

Over the last few decades, the UK Ladybird Survey dataset has been used to address a range of ecological questions. Many of the studies have sought to enhance the understanding of *H. axyridis* including patterns of spread^7,8^, impacts on native ladybirds^9^ and interactions with other drivers of biodiversity loss^10^. Additionally, distribution trends of ladybirds in the UK have been assessed for publication in the ladybird atlas^11^, field guide^12^, and as part of a broader analysis of long-term trends of invertebrates, bryophytes, and lichens^13^.

It is widely acknowledged that the traits of species can inform understanding of the mechanisms of biodiversity loss in response to drivers of change including climate change and land-use change. Comont *et al*.^14^ used traits to explain the distribution patterns of ladybirds in Great Britain (GB), highlighting the importance of diet breadth in determining range size.

**Fig. 2:**
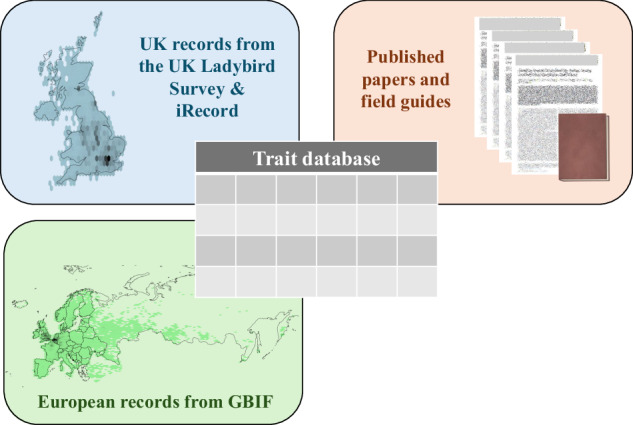
The data sources underlying the ladybird trait database. The various traits, ecological preferences and distribution metrics provided in this dataset have been determined through a combination of information from the literature, including published papers, the recently published Field Guide to the Ladybirds of Great Britain and Ireland^12^, biological records collected from the UK Ladybird Survey (www.coleoptera.org.uk/coccinellidae/home) and iRecord (www.irecord.org.uk) sourced through the National Biodiversity Network Atlas (www.nbnatlas.org), and biological records from across Europe from the Global Biodiversity Information Facility (www.gbif.org).

We have now updated and extended the dataset compiled by Comont *et al*.^14^ to include inconspicuous (micro) species of ladybirds within tribes Coccidulini and Scymnini and a number of new variables. Using published literature, occurrence records, and the recently published Field Guide to the Ladybirds of Great Britain and Ireland^12^ we have collated information on traits, ecological preferences and distribution for all ladybird species resident in the UK (Fig. 2).

## Methods

Using a range of published sources, a field guide^12^, and biological records collected for ladybirds from the UK Ladybird Survey and iRecord (www.irecord.org.uk; a website and mobile phone application for managing and sharing wildlife observations), we determined a comprehensive trait database for 48 species of ladybird that are considered resident in the UK. Ecological traits including information on ladybird species taxonomy and morphology (Table 1), distributions (Table 2), trends in occupancy over time (Table 3), diet preferences (Table 4), habitats (Table 5) and temperature indices and voltinism (Table 6) have been determined where possible for each species.

**Table 1:** A description of fields in the ladybird trait database: ladybird taxonomy and morphology.

Field	Description	Data type and value range	Unit
Genus	Scientific name, genus.	Character	NA
Species	Scientific name, species.	Character	NA
Common_name	English vernacular name of the species as presented in Roy & Brown^12^, The Field Guide to Ladybirds of Great Britain and Ireland.	Character	NA
Authority	Authority of the species name as presented in Roy & Brown^12^, The Field Guide to Ladybirds of Great Britain and Ireland.	Character	NA
Tribe	Tribe within which the species is placed.	Character	NA
Subfamily	Subfamily within which the species is placed.	Character	NA
Conspicuous	Species are split between two groups: Conspicuous (easily recognisable as ladybirds) and Inconspicuous (recognisable as ladybirds only by experts).	Categorical: Conspicuous or Inconspicuous	NA
Body_size_min	The minimum body size.	Numeric: range between 1.2 and 7	Millimetres
Body_size_max	The maximum body size.	Numeric: range between 1.5 and 8.5	Millimetres
Polymorphism_melanic	Indication of whether the melanic polymorphisms are common, rare or not present.	Categorical: common, rare, no	NA
Polymorphism_spot	Indication of whether the spot-based polymorphisms are common, rare or not present.	Categorical: common, rare, no	NA

Where possible, this information has been supplied for each of the 48 species. “Field” describes the column name given in the database. A description of the data provided under that field is included as well as the data type, the range of the values within that field, and the units where applicable.

**Table 2:** A description of fields in the ladybird trait database: ladybird distribution.

Field	Description	Data type and value range	Unit
UK_native	Indication of whether the species is native (1) or non-native (0) in the United Kingdom.	Categorical: 0 or 1	0 = non-native, 1 = native
n_records	The number of records in the processed dataset including records from the UK Ladybird Survey and iRecord from between 1970 and 2023.	Numerical: range between 1 and 93,297.	Number of records
First_yr	The year of the first record in the period 1970 to 2023.	Numerical: range between 1970 and 2016.	Year
Most_recent_yr	The year of the most recent record in the period 1970 to 2023.	Numerical: range between 1996 and 2023.	Year
nrecs_England	The total number of records for England across the dataset.	Numerical: range between 1 and 89,477.	Number of records
Present_England	Indication of whether the species is present (1) or not present (0) in England.	Categorical: 1 only	0 = not present, 1 = present
nrecs_Wales	The total number of records for Wales across the dataset.	Numerical: range between 0 and 3,214.	Number of records
Present_Wales	Indication of whether the species is present (1) or not present (0) in Wales.	Categorical: 0 or 1	0 = not present, 1 = present
nrecs_Scotland	The total number of records for Scotland across the dataset.	Numerical: range between 0 and 4,870.	Number of records
Present_Scotland	Indication of whether the species is present (1) or not present (0) in Scotland.	Categorical: 0 or 1	0 = not present, 1 = present
nrecs_NorthernIreland	The total number of records for Northern Ireland across the dataset.	Numerical: range between 0 and 703.	Number of records
Present_NorthernIreland	Indication of whether the species is present (1) or not present (0) in Northern Ireland.	Categorical: 0 or 1	0 = not present, 1 = present
n_10k	The number of unique 10 km grid cells based on the Ordnance Survey National Grid reference system that records of the species were present within. This includes the whole of the UK.	Numerical: range between 1 and 2,104.	Number of unique 10 km grid cells.
n_vicecounties	The number of unique vice counties (Great Britain and Northern Ireland) that records of the species were present within.	Numerical: range between 1 and 119.	Number of unique vice counties.

Further detailed text in the legend for Table 1 also applies here.

**Table 3:** A description of fields in the ladybird trait database: ladybird trends in occupancy over time.

Field	Description	Data type and value range	Unit
Longterm_trend	The annual growth rate (% change per year) to get from the occupancy estimate in LT_first to the occupancy estimate in LT_last. These values have been rounded to 3 decimal places.	Numerical: range between −4.674 and 44.950.	Percentage change in occupancy per year
LT_trend_lower	Lower 95% credible interval for the long-term trend (based on 999 iterations). These values have been rounded to 3 decimal places.	Numerical: range between −5.443 and 36.436.	Percentage change in occupancy per year
LT_trend_upper	Upper 95% credible interval for the long-term trend (based on 999 iterations). These values have been rounded to 3 decimal places.	Numerical: range between −4.097 and 54.772.	Percentage change in occupancy per year
LT_first	First year that the long-term trend is based on. For most species this is 1970 but there are exceptions where species have arrived in later years.	Numerical: range between 1970 and 2002.	Year
LT_last	Final year that the long-term trend is based on. This is 2018 for all species.	Numerical: 2018	Year
Shortterm_trend	The annual growth rate calculated as the difference between the first and last year of the 10-year period ending in 2018. See methods. These values have been rounded to 3 decimal places.	Numerical: range between −10.924 and 20.540.	Percentage change in occupancy per year
ST_trend_lower	Lower 95% credible interval for the short-term trend (based on 999 iterations). These values have been rounded to 3 decimal places.	Numerical: range between −21.911 and 10.504.	Percentage change in occupancy per year
ST_trend_upper	Upper 95% credible interval for the short-term trend (based on 999 iterations). These values have been rounded to 3 decimal places.	Numerical: range between −2.657 and 35.186.	Percentage change in occupancy per year
ST_first	First year that the short-term trend is based on. For species where short-term trends were possible, this is 2009.	Numerical: 2009	Year
ST_last	Final year that the short-term trend is based on. This is 2018 for all species.	Numerical: 2018	Year

Further detailed text in the legend for Table 1 also applies here.

**Table 4:** A description of fields in the ladybird trait database: ladybird diet preferences.

Field	Description	Data type and value range	Unit
Foodguild_Adelgidae	Indicates whether this food guild is dominant (1), occasionally recorded (0.5) or is unrecorded (0) for the ladybird species.	Categorical: 0, 0.5 or 1	NA
Foodguild_Aleyrodidae	Indicates whether this food guild is dominant (1), occasionally recorded (0.5) or is unrecorded (0) for the ladybird species.	Categorical: 0, 0.5 or 1	NA
Foodguild_Aphididae	Indicates whether this food guild is dominant (1), occasionally recorded (0.5) or is unrecorded (0) for the ladybird species.	Categorical: 0, 0.5 or 1	NA
Foodguild_Coccidae	Indicates whether this food guild is dominant (1), occasionally recorded (0.5) or is unrecorded (0) for the ladybird species.	Categorical: 0, 0.5 or 1	NA
Foodguild_Diaspididae	Indicates whether this food guild is dominant (1), occasionally recorded (0.5) or is unrecorded (0) for the ladybird species.	Categorical: 0, 0.5 or 1	NA
Foodguild_Pseudococcidae	Indicates whether this food guild is dominant (1), occasionally recorded (0.5) or is unrecorded (0) for the ladybird species.	Categorical: 0, 0.5 or 1	NA
Foodguild_Psyllidae	Indicates whether this food guild is dominant (1), occasionally recorded (0.5) or is unrecorded (0) for the ladybird species.	Categorical: 0 or 0.5	NA
Foodguild_Plants	Indicates whether this food guild is dominant (1), occasionally recorded (0.5) or is unrecorded (0) for the ladybird species. Note that this also includes pollen.	Categorical: 0, 0.5 or 1	NA
Foodguild_Fungi	Indicates whether this food guild is dominant (1), occasionally recorded (0.5) or is unrecorded (0) for the ladybird species.	Categorical: 0, 0.5 or 1	NA
Foodguild_Mites	Indicates whether this food guild is dominant (1), occasionally recorded (0.5) or is unrecorded (0) for the ladybird species.	Categorical: 0, 0.5 or 1	NA
Foodguild_Other	Indicates whether this food guild is dominant (1), occasionally recorded (0.5) or is unrecorded (0) for the ladybird species. This includes Apidae + Chrysomelidae + Chrysopidae + Cicadellidae + Coccinellidae + Curculionidae + Eriococcidae + Insect_other + Lepidoptera_unspecified + Liviidae + Matsucoccidae + Monophlebidae + Noctuidae + Nymphalidae + Pamphiliidae + Phylloxeridae + Psocodea + Pyralidae + Rhodobacteraceae + Tenthredinidae + Thripidae + Thrips_unspecified.	Categorical: 0, 0.5 or 1	NA
nfood_recs	The total number of feeding records, i.e. papers recording the ladybird feeding on a species, species from all guilds summed. The data are from field and laboratory studies so include field records of ladybirds observed feeding, plus accepted foods reported in laboratory studies.	Numerical: Between 1 and 111.	NA
Foodguild_confidence	Indicates the level of confidence in the Foodguild values, based on the number of feeding records for each ladybird.	Categorical: low, medium or high	Level of confidence: High = over 10 feeding records (all foods combined) for the ladybird, medium = 6 to 10 feeding records, low = 1 to 5 feeding records
nfamilies_diet	The number of prey families recorded for the ladybird.	Numerical: Between 1 and 16	NA

Further detailed text in the legend for Table 1 also applies here.

**Table 5:** A description of fields in the ladybird trait database: ladybird habitats.

Field	Description	Data type and value range	Unit
Forest_woodland	Indicates whether the species is often found (1), occasionally found (0.5) or is absent (0) within this habitat type.	Categorical: 0, 0.5 or 1	NA
Shrubland	Indicates whether the species is often found (1), occasionally found (0.5) or is absent (0) within this habitat type.	Categorical: 0, 0.5 or 1	NA
Native_grassland	Indicates whether the species is often found (1), occasionally found (0.5) or is absent (0) within this habitat type.	Categorical: 0, 0.5 or 1	NA
Wetlands_inland	Indicates whether the species is often found (1), occasionally found (0.5) or is absent (0) within this habitat type.	Categorical: 0, 0.5 or 1	NA
Marine_intertidal	Indicates whether the species is often found (1), occasionally found (0.5) or is absent (0) within this habitat type.	Categorical: 0, 0.5 or 1	NA
Marine_coastal_supratidal	Indicates whether the species is often found (1), occasionally found (0.5) or is absent (0) within this habitat type.	Categorical: 0, 0.5 or 1	NA
Artificial_terrestrial	Indicates whether the species is often found (1), occasionally found (0.5) or is absent (0) within this habitat type.	Categorical: 0, 0.5 or 1	NA
Other	Indicates whether the species is often found (1), occasionally found (0.5) or is absent (0) within any other habitat type not already specified.	Categorical: 0, 0.5 or 1	NA
n_habitats_weighted	Number of IUCN habitats accounting for use level i.e. sum of habitat columns.	Numerical: range between 1 and 6.	Number of habitats the species is present in weighted by preference
n_habitats	Number of IUCN habitats i.e. number of unique habitats the species is found in.	Numerical: range between 1 and 6.	NA

Further detailed text in the legend for Table 1 also applies here.

**Table 6:** A description of fields in the ladybird trait database: ladybird temperature indices and voltinism.

Field	Description	Data type and value range	Unit
Larvae_first	Month of the first larval record for the species.	Numerical: range between 1 and 7.	Month number where January = 1.
Larvae_last	Month of the last larval record for the species.	Numerical: range between 6 and 12.	Month number where January = 1.
Adult_first	Month of the first adult record for the species.	Numerical: range between 1 and 5.	Month number where January = 1.
Adult_last	Month of the last adult record for the species.	Numerical: range between 5 and 12.	Month number where January = 1.
Peak_larvae	The month in which larval records peak.	Numerical: range between 5 and 9.	Month number where January = 1.
Peak_adult1	The month in which adult records peak.	Numerical: range between 4 and 9.	Month number where January = 1.
Peak_adult2	If relevant, the month in which adult records peak for a second time.	Numerical: range between 7 and 10.	Month number where January = 1.
Max_month	The month of the year with the highest number of records of the species across the UK.	Numerical: range between 4 and 10.	Month number where January = 1.
Max_month_EU	The month of the year with the highest number of records of the species across Europe.	Numerical: range between 4 and 9.	Month number where January = 1.
Voltinism_min	The minimum number of generations that a species is known to have within a year.	Numerical: range between 1 and 2.	NA
Voltinism_max	The maximum number of generations that a species is known to have within a year.	Numerical: range between 1 and 3.	NA
STI_UK_mean	The mean annual temperature across the species’ range using UK records only and temperatures from all months of the year.	Numerical: range between 8.31 and 10.29.	°C
STI_UK_peak	The mean annual temperature across the species’ range using UK records only and temperatures from the month with the most records.	Numerical: range between 7.73 and 16.86.	°C
STI_Europe_mean	The mean annual temperature across the species’ range using European records only and temperatures from all months of the year.	Numerical: range between 3.01 and 11.01.	°C
STI_Europe_peak	The mean annual temperature across the species’ range using European records only and temperatures from the month with the most records.	Numerical: range between 7.84 and 18.73.	°C

Further detailed text in the legend for Table 1 also applies here.

Previously, ladybird trait data were collated and analysed to understand the distribution, colonisation and extinction of ladybirds in Great Britain^10,14^, however, the traits dataset was not openly available. Here, we have added new variables to the dataset, expanded the dataset to include the inconspicuous ladybirds (those in tribes Coccidulini and Scymnini, i.e. species generally not immediately recognised by non-experts as ladybirds) where possible, reviewed the information on the traits from previous publications^10,14^ and made the data openly available^15^.

For the 48 species (27 conspicuous ladybirds and 21 inconspicuous ladybirds), we have collated information on body size, polymorphism (Table 1), UK native status including the number of records and presence in England, Scotland, Wales and Northern Ireland since 1970, the number of 10 km grid squares and vice counties occupied (Table 2), long- and short-term trends in species occupancy (Table 3), feeding preferences and the number of and detail on the families recorded in the species’ diet (Table 4), classifications of habitats in which species are found (Table 5), months of the first larval and adult records, peak months of activity for larvae and adult populations based on species records, the month with the most adult records, the number of generations per year (voltinism), and UK and European-scale species temperature indices (Table 6). Images of species have not been provided within the database itself, however, images of both conspicuous (https://coleoptera.org.uk/coccinellidae/conspicuous-ladybirds) and inconspicuous species https://coleoptera.org.uk/coccinellidae/inconspicuous-ladybirds) are available via the UK Ladybird Survey webpages.

### Determination of each variable

**Fig. 3:**
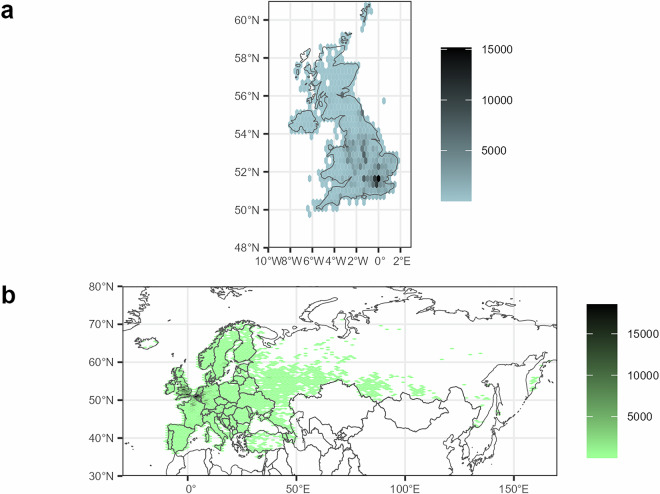
The distribution and number of records of the 48 ladybird species in (**a**) the United Kingdom and (**b**) Europe. Records were supplied by the UK Ladybird Survey via UKCEH and iRecord from the NBN Atlas for the UK, and from GBIF for Europe. See methods for detail on the data filtering and cleaning process.

Each variable was determined using information either from the literature, from the Field Guide to the Ladybirds of Great Britain and Ireland^12^ or using biological records for the UK from the UK Ladybird Survey (provided directly by UKCEH) and iRecord which were downloaded from the National Biodiversity Network (NBN) Atlas^16^, or for Europe from the Global Biodiversity Information Facility^17^ (Fig. 3).

The biological records from both the UK Ladybird Survey and iRecord represent verified occurrences of species at a known time and location and combined consist of 376,012 records from between 1816 and 2024 before processing. UK Ladybird Survey data were supplied directly by the Biological Records Centre at UKCEH and iRecord records were available via the NBN Atlas^16^. For our purposes, we used records from the UK (England, Scotland, Wales and Northern Ireland only, excluding the Isle of Man and the Channel Islands) that were identified to the species level, where complete information on the date of the record was known to the day for the years 1970 to 2023. Records before 1970 were excluded due to their small number and records in 2024 were only available up to February. We also removed the small number of records where the precision of the supplied grid references was at 2,000 and 5,000 m as it was not possible to convert these to latitude and longitude using the *gr2gps*_*latlon* function available from the *sparta* R package^18^. These data included information on 53 ladybird species including five species that are not included in our trait database (*Cryptolaemus montrouzieri, Exochomus nigromaculatus, Oenopia conglobata, Rodolia cardinalis, Scymnus rubromaculatus*). These species are not currently considered resident in the UK, and sightings are thought to be the result of unintentional introductions into the wild or are vagrants from Europe. Each had only between 1 and 6 records. After filtering for these requirements, we were left with 343,503 records. This dataset was then used to determine several of the variables as detailed below. All record-based variables were determined using R version 4.4.1^19^.

#### Body size

Published sources were checked for body size estimates (see database file “sources.csv” for a list of sources). The entire range of values from the sources was included as a minimum and maximum body size estimate in millimetres.

#### Polymorphism

Two types of polymorphism are present in ladybirds: (1) the melanic form, where individuals show variation in colour, and (2) the spotted form, where individuals show variation in spot patterns^12,20^. Here, we have indicated whether each form for each species is common, rare, or not present.

#### UK native

A number of species that are now resident in the UK are non-native species. We have therefore indicated whether (1) or not (0) each species is considered as native based on information from Roy & Brown^12^.

#### Record summaries

Using the biological records, for each species the total number of records, the year of the first record, and the year of the most recent record were determined.

#### Country level presence

Depending on the distribution of each species they may or may not be present within each of England, Wales, Scotland and Northern Ireland. We have therefore indicated whether the species is present within each of those countries based on whether there was at least one record from that country in the UK dataset. The number of records found within each country was also determined.

#### Number of 10-kilometre grid squares

Ladybird records for each species were overlaid onto a grid of 10 km cells representing the Ordnance Survey National Grid reference system (available from Ordnance Survey via Github^21^) and the number of unique 10 km grid cells within which records were present was determined for each species. The set of grid cells covered Great Britain and Northern Ireland.

#### Number of vice-counties

For each species, we determined the number of unique vice-counties within which records are found using polygons of the British vice-counties available via the Biological Records Centre resources Github^22^ and of the Irish vice-counties also available via Github (https://github.com/SK53/Irish-Vice-Counties).

#### Long-term and short-term trends

**Fig. 4:**
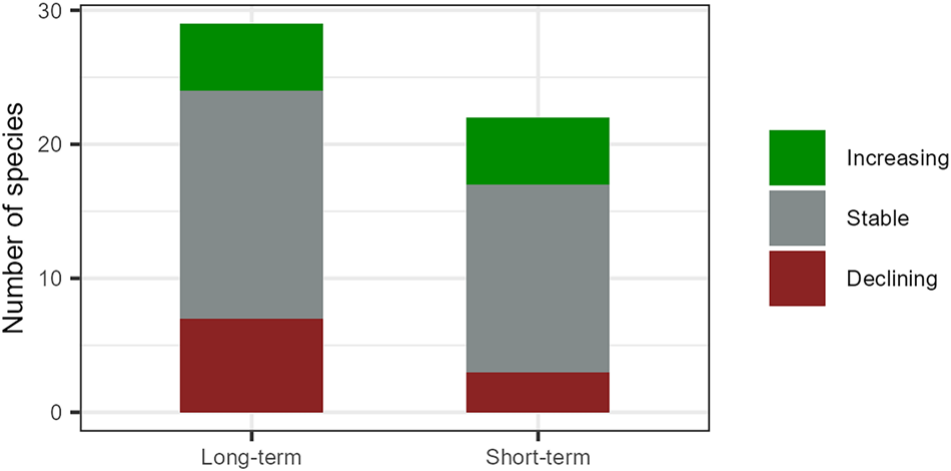
The number of ladybird species whose UK trends are increasing, stable or declining over the long- and short-term. Increasing and declining species were classified based on whether the 95% credible intervals were both greater than or less than zero, respectively. Long-term trends were available for 29 of the 48 species, short-term trends were available for 22 of the 48 species.

Trend estimation was undertaken as part of the DRUID project (https://druidproject.org.uk/), and so involved the use of a slightly different dataset to that used to determine the other variables presented in this database. Trends in species occupancy (or the proportion of occupied sites) were determined from biological records collected via the UK Ladybird Survey and iRecord plus a number of supplementary datasets^23–26^. Trends were calculated for the United Kingdom, however data from Northern Ireland was limited for most species. Data collation and preparation followed that of previous studies^27,28^, where presence only species occurrence records were filtered to day-resolution at the 1 km grid square scale or finer (reformatted to the 1 km grid square scale). All species records made between 1970 and 2021 were included in an occupancy model analysis, however trends were calculated with the final year set to 2018, discussed below. First, biological records were used to determine annual estimates of species occupancy for the years 1970 to 2021, or more recently if the first record for the given species was after 1970, following the Bayesian occupancy modelling approach outlined in Outhwaite *et al*.^27^. Furthermore, we dropped annual occupancy estimates where the model failed to converge (rhat > 1.1), meaning the first year of the trend calculation was determined as the first converged year of or after the first record of the given species. Using the distributions of estimates of annual occupancy from the models, species trends were calculated as the annual growth rate (percentage change per year) between the first and last year, for both the long-term and the short-term. For the long-term trends, the first year was 1970 (or later if the species arrived after this point) and the last year was 2018. For the short-term trends, the first year was 2009 and the last year was 2018. The first and last years are provided for reference in the database alongside the long-term and short-term trends and the upper and lower 95% credible intervals of the trend estimates. Although records up to 2021 were used to determine the occupancy estimates, trends were only calculated up to the year 2018. This was because trends calculated to later years showed the potential influence of changes in recorder effort due to the release of the Field Guide to the Ladybirds of Great Britain and Ireland^12^ in 2018. This particularly affected the inconspicuous species which would have been challenging for amateurs to identify before publication of the field guide. Note that some species are missing short-term trends. This is because the estimate for the first year in the model did not converge and so would be considered unreliable. Long-term trends could be calculated for 29 of the 48 species and short-term trends for 22 of the 48 species (Fig. 4).

#### Feeding guild and number of prey families

A literature search was conducted to identify records of prey species for each ladybird (larval and/or adult stages) (see database file “prey_list.csv” for a list of included prey and database file “sources.csv” for a list of sources). The family of each prey species was assigned with reference to GBIF taxonomy (www.gbif.org). Based on this information, eleven feeding guilds were populated based on whether they were identified as a dominant prey group (i.e. in most cases representing at least 50% of total feeding records), an occasional prey group (i.e. there was at least one feeding record, but it represents a minority of total feeding records) or not recorded as a prey group for each species. The total number of different prey families recorded per ladybird species is shown. Prey families include invertebrates, plants and fungi. There are potentially biases in prey family numbers caused by differing levels of interest and knowledge of the different ladybirds and their prey species. We therefore assigned a confidence level to each species based on the amount of information available, where high = over 10 feeding records (all foods combined) for the ladybird, medium = 6 to 10 feeding records, low = 1 to 5 feeding records. Whilst this gives an indication of our confidence, a food source may be inflated by the same author publishing multiple papers reporting similar results.

#### Habitat association and number of habitats

Species were assigned to the Level 1 habitats of the IUCN Habitat Classification Scheme v3.1^29^. Each species was classified as present (assigned value of 1, i.e. commonly recorded in a habitat), occasionally present (0.5, i.e. infrequently recorded), or absent (0, i.e. very rarely or never recorded) within each habitat based on information from the Field Guide to the Ladybirds of Great Britain and Ireland^12^ in conjunction with expert knowledge of co-authors PMJB and HER. The weighted number of habitats was calculated as the number of habitats, accounting for whether the species is primarily present or occasionally present in that habitat. The total number of habitats was calculated as the number of unique habitats the species is present in, irrespective of whether it is common there or only found occasionally.

#### First, last, maximum and peak month of species records

For both the larval and adult form of each species we determined the first month in the year with records, the last month in the year with records, and the month with the most records of that species. Larval months were determined from an unpublished dataset of larval stage records from the Biological Records Centre which consisted of 5,644 records from between 1929 and 2021. Note that some species have very few larval records and so these values may not fully represent reality. Larval records were not available for the inconspicuous species and so first, last and peak months have not been determined for the larval stage. For species with sparse larval records, the peak month is unavailable as there are equal numbers of larval records in multiple months.

Adult months were determined using the processed set of biological records from the UK Ladybird Survey and iRecord described above. Here, the peak months were determined using the *peaks* function in the *splus2R* R package^30^ with the *span* variable set to 5. Note that in some cases species may have two peak months. Histograms of the records were also generated, and the peaks checked against them. Where peaks generated were likely due to a low number of records, rather than being genuine peaks in activity, these were assessed by the authors and where appropriate, set to NA. For the adult form, the month with the maximum number of records was also determined from the processed records. This was carried out for both the UK and European-scale datasets so that species temperature indices could be determined for the month of peak activity (see later section).

#### Voltinism

This describes the number of generations that each species is known to complete within a year. This can be variable depending on the conditions of that year, the region and whether wild or laboratory populations were examined, and so a minimum and maximum has been provided where the data are available. This information was collated from published sources (see database file “sources.csv” for a list of sources). For a number of species this information was not available from any source. We have therefore assumed, in those cases, a minimum number of generations of 1 and have set the maximum to NA.

#### Species temperature indices (STIs)

This metric gives an indication of the long-term temperatures that a species has experienced over its range and is used to represent a species’ temperature preference (e.g.^31,32^). Here, the species temperature index is calculated as the mean annual temperature across a species range (the UK range and the European range separately), based on available biological records and monthly mean temperature data from WorldClim historical climate data version 2.1^33^. Temperature data were at 10-minute resolution and provide average monthly temperature estimates for the period 1970 to 2000.

For the UK-based STIs, we used the processed records from the UK Ladybird Survey and iRecord and determined two STIs for each species, one based on all months of the year, and one based on just the month with the most records (representing a peak period of activity). To calculate the mean STI, the temperature values across all months were extracted for the location of each of the records of the species and the average taken. Similarly, for the peak STI, temperature values from just the month with the most records of that species were extracted for the location of each record and the average taken. In the database, these values have been rounded to two decimal places.

For the Europe-based STIs, data on ladybird occurrences were downloaded from GBIF^17^. The data download and partial processing used the *rgbif* R package^34^. The GBIF taxon keys for each of the 48 species were determined and used to download relevant records. Records were downloaded with the following specifications: the basis of record was “human observation”, “observation” or “machine observation”, the coordinates of the record were known, there were no known geospatial issues, the occurrence status was “present”, and the records were from 1970 onwards. The data download consisted of 1,584,603 records. Further filtering and cleaning of the data was undertaken using the *CoordinateCleaner* R package^35^ to ensure that incorporated records were as reliable as possible. This included: records with a coordinate precision more than 0.01 or coordinate uncertainty more than 10000 were removed, records with a known default value in the coordinate uncertainty column (including 301, 3036, 999 and 9999) were removed, records where the latitude and longitude values were both 0 were removed, records where the month of the record was unknown were removed (this is required for the determination of the peak month of records), records where the location was within 2 km of a country centroid were removed, records within 2 km of a zoo or herbarium were removed, records in the sea were removed, and duplicated locations were removed. Finally, we only included records that were from European countries. This resulted in a dataset consisting of 631,289 records for the 48 species of interest. Using the dataset, we were then able to determine the mean and peak STIs as detailed above.

### Missing information

Where information is unknown for a species-variable combination, cells in the database are set to NA. Some species, particularly the inconspicuous ladybirds, are less well studied than others and so information may be limited. Since the release of the Field Guide to the Ladybirds of Great Britain and Ireland^12^ in 2018, there is greater familiarity with the inconspicuous ladybirds amongst the recording community than before. Furthermore, the illustrations within the field guide provided, for the first time, an easily accessible resource for identification. Additional online resources (for example https://www.andrewjewels.com and https://commonbynature.com/inconspicuous-ladybirds) have since been published and undoubtedly increased the interest in recording these small beetles which will ultimately increase our understanding of their ecology and traits. We hope that this trait database will be updated in the future to reflect any increase in knowledge.

## Data Records

The data are available open access from the Environmental Information Data Centre repository (EIDC)^15^. The dataset is organised into four files:

1. ladybird_traits.csv – This is a csv file containing the trait database. It consists of 49 rows and 74 columns. The first row details the column names (detailed also in Tables 1–6). Subsequently, there is a row for each of the 48 species.
2. metadata.csv – This is a csv file containing the information in Tables 1–6 as well as some information on the sources of information for each column. This file has 75 rows and 7 columns.
3. sources.csv – This is a csv file containing the list of sources used to compile information on various variables included in the database. It consists of 277 rows and 3 columns. The first row contains the header variables. Subsequent rows detail the list of sources, the variables they were used to support and available information to access the source where available (i.e. DOIs).
4. prey_list.csv – This is a csv file containing the list of prey families identified for each ladybird species from the data sources. It consists of 640 rows and 3 columns.

## Technical Validation

### Record validation

Records from both the UK Ladybird Survey and iRecord are verified by UK Ladybird Survey scheme organisers and selected expert verifiers. The validation process involves checks to ensure start and end dates are valid dates, and that the location is within a grid reference that contains land. Records were filtered so that only those with a known date (from 1970 onwards) and location were included.

### Variable validation

Derived traits, i.e. habitat and those related to phenology, underwent expert validation by PMJB and HER. Peak months of records calculated using the *peaks* function in *splus2R* package^30^ were compared visually with histograms of records per month to confirm appropriate parameter setting and to correct those influenced by low record number.

### Recording intensity

There is high temporal variability in recording intensity. Species trends, therefore, were only estimated using data up to 2018 to avoid the influence of increased recording intensity following the publication of the Field Guide to the Ladybirds of Great Britain and Ireland^12^ in 2018.

### Inconspicuous species

For some of the inconspicuous species, many of the data are still unavailable due to differences in detectability and recorder effort, e.g. larval stage information. Some of the columns for these species, therefore, have been recorded as NA as reliable estimates are not yet possible due to the scarcity of the data.

### The Field Guide to the Ladybirds of Great Britain and Ireland

Information within the Field Guide to the Ladybirds of Great Britain and Ireland^12^ was compiled from a variety of sources including the UK Ladybird Survey dataset, the Naturalists' Handbook^20^ and the Ladybirds of Britain and Ireland^11^. These publications in turn are based on compilations of information within reviews of ladybirds such as ref. ^36^ but also other books including ref. ^37^. Notes outlining the details of the species accounts within ref. ^12^ and ref. ^11^ provide further details. We are therefore confident that the information provided within the field guide is the most up to date and accurate source of data available to underpin the trait database.

## Data Availability

The data are available open access from the Environmental Information Data Centre repository^15^ (10.5285/18cdeee4-38cf-4d15-a141-a99a53e17095). The dataset consists of four csv files as described in the Data Records section.

## Code availability

The code used to download GBIF data, process all record-based data, determine record-based variables and create figures presented in this paper is available from https://github.com/CharlieOuthwaite/LadybirdTraits. Note that a GBIF account is required to download data from GBIF.
